# Correction

**DOI:** 10.1093/ckj/sfae344

**Published:** 2024-11-20

The originally published versions of the below-listed manuscripts inadvertently omitted a link to their accompanying videos:

https://doi.org/10.1093/ckj/sfae236

https://doi.org/10.1093/ckj/sfae199

This has been corrected.

